# Pancreatic Organoids for Regenerative Medicine and Cancer Research

**DOI:** 10.3389/fcell.2022.886153

**Published:** 2022-05-03

**Authors:** Joan Casamitjana, Elisa Espinet, Meritxell Rovira

**Affiliations:** ^1^ Department of Physiological Science, School of Medicine, University of Barcelona (UB), L'Hospitalet de Llobregat, Barcelona, Spain; ^2^ Pancreas Regeneration: Pancreatic Progenitors and Their Niche Group, Regenerative Medicine Program, Institut D’Investigació Biomèdica de Bellvitge (IDIBELL), L’Hospitalet de Llobregat, Barcelona, Spain; ^3^ Program for Advancing the Clinical Translation of Regenerative Medicine of Catalonia (P-CMR[C]), L’Hospitalet de Llobregat, Barcelona, Spain; ^4^ Department of Pathology and Experimental Therapy, School of Medicine, University of Barcelona (UB), L’Hospitalet de Llobregat, Barcelona, Spain; ^5^ Molecular Mechanisms and Experimental Therapy in Oncology Program (Oncobell), Institut D’Investigació Biomèdica de Bellvitge (IDIBELL), L’Hospitalet de Llobregat, Barcelona, Spain

**Keywords:** organoids, pancreas, PDAC, diabetes, regenerative medicine, personalized medicine

## Abstract

In recent years, the development of *ex vivo* organoid cultures has gained substantial attention as a model to study regenerative medicine and diseases in several tissues. Diabetes and pancreatic ductal adenocarcinoma (PDAC) are the two major devastating diseases affecting the pancreas. Suitable models for regenerative medicine in diabetes and to accurately study PDAC biology and treatment response are essential in the pancreatic field. Pancreatic organoids can be generated from healthy pancreas or pancreatic tumors and constitute an important translational bridge between *in vitro* and *in vivo* models. Here, we review the rapidly emerging field of pancreatic organoids and summarize the current applications of the technology to tissue regeneration, disease modelling, and drug screening.

## Introduction

**FIGURE 1:**
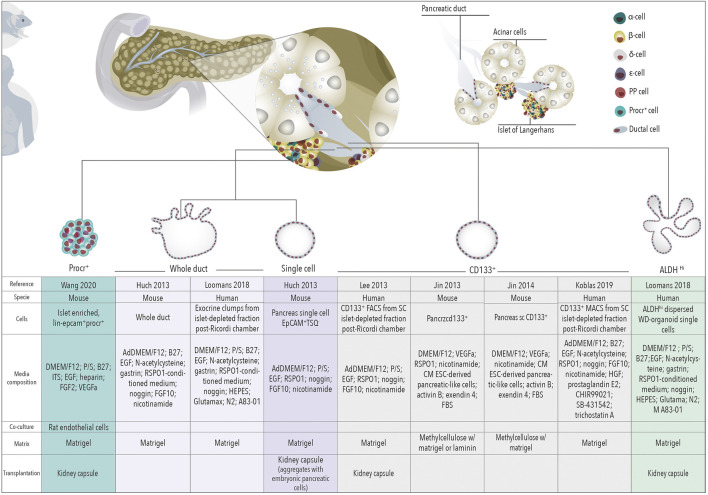
Organoids for regenerative medicine. Schematic representation of organoids derived from different cells of the healthy pancreas towards regenerative medicine for β cell replacement.

The pancreas is an endoderm derived gland with an endocrine and an exocrine compartment. The endocrine compartment is formed by hormone producing cells that regulate blood glucose levels. Within the most abundant endocrine cells we find insulin producing β cells, glucagon producing α cells, somatostatin producing δ cells, and pancreatic polypeptide producing PP cells (Figure 1). They are organized in highly vascularized clusters termed Islets of Langerhans, which comprise ∼1–2% of the organ (Wang et al., 2016; Marsee et al., 2021). The rest of the gland displays an exocrine function. Exocrine pancreas is mainly formed by acinar cells, organized in acini, which produce and secrete digestive enzymes that are transported into the duodenum through an intricate network of tubules formed by the other exocrine cell type, the ductal cells, which secrete bicarbonate to neutralize stomach acidity (Grapin-Botton, 2005) (Figure 1).

Dysregulation of either the endocrine or the exocrine pancreas results in two major diseases: diabetes mellitus and pancreatic ductal adenocarcinoma (PDAC), respectively. Diabetes is a metabolic disorder characterized by loss or disfunction of pancreatic β cells. There are 537 million adults (20–79 years) living with diabetes and its prevalence is increasing and predicted to rise to 643 million by 2030 (IDF Diabetes Atlas 10th edition), making diabetes a major public health challenge worldwide. Current treatments for insulin-dependent diabetic people are based on multiple insulin injections coupled with regular blood glucose monitoring (Melton, 2021). Although there have been major advances in pharmacogenetic findings of antidiabetic agents, patients and their families still don’t live free from the constant burden of monitoring blood glucose levels, insulin pumps, diet and exercise. Importantly, transplantation of cadaveric islets arose as a promising therapy option leading to impressive results on insulin independence (Hering et al., 2016; Lablanche et al., 2018; Shapiro et al., 2000). However, access to islets is limited by the number of deceased donors. Additionally, transplantation requires the life-long induced immunosuppression of the patient (Vantyghem et al., 2019). Together, these preclude the widespread use of cadaveric islet transplantation to treat diabetes. Alternative cell replacement therapies to find an unlimited source of β cells have been investigated for the last couple of decades. On the one hand, protocols have been developed to induce endocrine cells differentiation, specially β cells, from human embryonic stem cells (ESC) (Balboa et al., 2022; Rezania et al., 2014) and induced pluripotent stem cells (iPSC) (Nostro et al., 2011). These cells have recently been reported to be able to produce insulin in human patients using macroencapsulation systems in two phase I/II clinical trials (clinicaltrials.gov). The first reports of one of the trials have been recently released showing device safety and insulin secretion from engrafted pluripotent stem cell-derived pancreatic progenitor cells in patients with type 1 diabetes (Ramzy et al., 2021; Shapiro et al., 2021). On the other hand, multiple adult pancreatic cell types have been identified to be able to give rise to β cells and are being investigated as inducible endogenous progenitors (Afelik and Rovira, 2017), specially using organoid cultures.

Pancreatic ductal adenocarcinoma (PDAC) is one of the deadliest types of cancer with a 5-year survival rate of approximately 9%. PDAC is the third cause of cancer related death in the United States and Europe behind lung and colorectal cancer and its incidence is estimated to increase in the next years (Siegel et al., 2020). The main reasons for such a devastating outcome are late detection and poor response to treatment. This highlights the urgent need for appropriate models to study tumor onset and for platforms that allow the investigation of more efficacious therapies. Unfortunately, genetic mouse models do not fully recapitulate the complex human scenario and the study of human early disease is challenging as the majority of patients are diagnosed in an advanced disease stage. Similarly, access to large amounts of human primary material for direct drug testing studies is typically limited as only 20% of PDAC patients are eligible for resection and fine needle biopsies from non-resectable patients result in recovery of too few tumor cells. Organoid models appear as an attractive model in the field of pancreatic cancer research to overcome these limitations.

Organoids are 3D *in vitro* models of self-renewing cells, which spontaneously self-organize into structures with similarities to their corresponding *in vivo* tissue (Marsee et al., 2021; Box 1). Organoids can be used to study healthy or diseased tissue and can be generated from embryonic progenitors, adult-derived stem/progenitor cells, tumor samples or differentiated from pluripotent stem cells (iPSC/ESC). Although the capacity of pancreatic organoids derived from healthy tissue to recapitulate tissue differentiation and architecture is limited, pancreatic organoids open a window of opportunity to develop regenerative medicine therapies for diabetes and disease modeling. Additionally, PDAC derived organoids can be used for the development of personalized medicine therapies and drug screening. In this review we highlight the advances made in the development and use of pancreatic organoids in these topics and discuss the future challenges in this fast-developing field.

## Healthy Tissue Derived Organoids for Regenerative Medicine

Highly proliferative tissues such as intestine and skin harbor a significant pool of progenitors (Gonzales and Fuchs, 2017; McCarthy et al., 2020), which comprise ∼5–8% of the tissue and allow its renewal in homeostatic conditions or upon injury (Gonzales and Fuchs, 2017; Guiu et al., 2019). Other tissues such as liver, display a low turn-over of cells in homeostasis and only specific cells proliferate upon injury to regenerate the damaged tissue (Campana et al., 2021). The pancreas does not display regenerative ability in homeostasis nor upon injury and therefore has limited capacity for regeneration, specially the endocrine compartment (Zhou and Melton, 2018). Although in the last couple of decades many laboratories have investigated the presence of pancreatic progenitors in the adult tissue to identify an unlimited source of β cells for replacement therapies, to date, most lineage tracing reports suggest that dedicated progenitors do not exist in this gland. Instead, some terminal differentiated pancreatic lineages, such as acinar, ductal and endocrine cells (Afelik and Rovira, 2017), have the ability to give rise to β cells under specific injury models pointing out at high pancreatic plasticity.

Acinar cell potential to give rise to β cells has been investigated showing none or extremely low efficiency in homeostasis and regenerative conditions (Pan and Wright, 2011). However, acinar cells display high plasticity or transdifferentiation capacity when compared to other pancreatic cells. Thus, acinar cells have been reprogrammed *in vivo* into insulin expressing cells upon lentiviral infection of three master regulators of endocrine differentiation, Neurog3, Pdx1, and MafA (Azzarelli et al., 2017). Recently it has been described that inflammation plays and important role in acinar to β transdifferentiation thus only when inflammation is attenuated, either by reducing the intensity of transcription factor expression or by depleting macrophages, the production of new β-like cells occur (Clayton et al., 2016). Moreover, *in vitro* murine and human acinar cells transdifferentiate into an embryonic-like phenotype (Houbracken et al., 2011; Pinho et al., 2011). Therefore, delineating acinar cell culture conditions may uncover acinar-to-β-cell transdifferentiation in the future.

Both acinar and ductal cells can initiate organoid cultures, therefore containing proliferative cells or cells able to enter the cell cycle. Nevertheless, only ductal cells are able to form organoids that can be expanded and maintained *in vitro* over time while acinar cells cannot (Huch et al., 2013). This implies that having proliferative capacity is not sufficient to efficiently form organoids. Cells should also require a certain degree of cellular plasticity *in vitro* which seem to be exclusive to ductal cells. As the capacity of organoids to expand over numerous passages proves the presence of stem/progenitor cells in the original preparation and has been linked to the existence of adult progenitors in other tissues, these results suggest that, in the pancreas, progenitors are likely located in the ductal compartment. Although, so far, the capacity of pancreatic organoids derived from healthy tissue to recapitulate tissue differentiation and architecture is limited, pancreatic organoids hold promise for the development of regenerative medicine therapies for diabetes and disease modelling.

### Human and Mouse Ductal Derived Organoids for Diabetes Treatment: A Promise Not Yet Fulfilled

Pancreatic organoids were first described by Clevers’ laboratory in 2013 (Huch et al., 2013), introducing pancreatic organoid technology as a putative unlimited source of induced-progenitors with the prospect of future differentiation into insulin secreting cells. The adult pancreas does not express the stem marker Lgr5 in homeostatic conditions (Sato et al., 2009). However, mouse pancreatic duct fragments were shown to initiate Lgr5 expression in RSPO1-based cultures followed by organoid formation (Huch et al., 2013). Organoids derived from mouse and human pancreatic tissue expressed ductal markers (*SOX9, KRT19, MUC1*) and only organoids expressing Sox9 were capable of long-term expansion (Huch et al., 2013; Boj et al., 2015). Together, these observations suggest that ductal cells *in vitro* display progenitor capacities. In line, organoids derived from EpCAM^+^-TSQ^-^ cells (i.e., non-endocrine epithelial cells) could be induced to differentiate into ductal as well as endocrine cells upon transplantation with embryonic E13 mouse or E14 rat pancreata under the kidney capsule of non-diabetic immunodeficient mice, although the efficiency of differentiation toward endocrine lineages was ∼5% (Huch et al., 2013). Later, lentivirus-mediated, doxycycline-inducible expression of Neurog3, Pdx1, and MafA in mouse pancreatic ductal organoids has been shown to generate cells that express insulin and resemble β-cells at the transcriptome level (Azzarelli et al., 2017). The combinatorial potential of these three transcriptional factors to induce endocrine differentiation was reported by Melton’s laboratory to induce transdifferentiation of acinar cells to β cells *in vivo* (Zhou et al., 2008). Finally, efficiency of organoid-derived β-like cell generation can be significantly enhanced by preventing phosphorylation of the Neurog3 protein and further augmented by conditions promoting differentiation (Azzarelli et al., 2017). This suggests that post-translational regulation of key regulators of endocrine differentiation can be exploited to enhance β-cell generation from organoids (Figure 1).

### ALDH Positive Ductal Derived Organoids

Pancreatic organoids have been subsequently derived from subpopulations of ductal cells defined by already identified progenitor markers. We showed that mouse ductal cells displaying high aldehyde dehydrogenase (ALDH) activity display progenitor features *in vitro*, in pancreatospheres cultures and upon transplantation into embryonic pancreas (Rovira et al., 2010). These cells demonstrated dramatic expansion in the setting of epithelial injury and pregnancy (Rovira et al., 2010; Ioannou et al., 2013; Socorro et al., 2017). ALDH^hi^ cells have indeed been identified in human fetal and adult pancreas (Loomans et al., 2018; Oakie et al., 2018). Recently, Koning’s laboratory (Loomans et al., 2018) showed that human ductal pancreatic cells displaying high ALDH activity can efficiently form organoids that can be expanded and maintained overtime. Interestingly, gene expression profiling revealed that ALDH^hi^ ductal expressing cells are closer to human fetal pancreatic tissue compared with adult pancreatic tissue (endocrine and exocrine). ALDH^hi^ derived organoids are able to differentiate into insulin expressing cells *in vitro* and upon being engrafted into the kidney capsule, although with low efficiency (∼1.5% of insulin positive cells) (Loomans et al., 2018) (Figure 1).

### CD133 Positive Ductal Derived Organoids

Similarly to ALDH, CD133 is a marker whose expression has been described as a cell surface marker in adult progenitor populations (Shmelkov et al., 2005; Li, 2013; Vassalli, 2019). CD133 is also expressed in a fraction of pancreatic ductal cells (Immervoll et al., 2008; Immervoll et al., 2011) and indeed, it has been used as surface marker to isolate ductal cells and assess their progenitor potential in cultures grown as monolayer colonies or spheres and their capacity to differentiate into endocrine cells *in vitro* (Oshima et al., 2007; Hori et al., 2008). CD133^+^ cells isolated from mouse adult pancreas form organoids (Jin et al., 2013; Jin et al., 2014; Wedeken et al., 2017) but these do not display long-term self-renewal potential and are heterogeneous. However, they displayed endocrine and exocrine differentiation capacity in presence of WNT ligand R-spondin1 (Jin et al., 2013; Jin et al., 2014). In humans, CD133^+^ isolated pancreatic cells form organoids with self-renewal potential (Lee et al., 2013) but their endocrine differentiation was only achieved upon adenoviral induction of the expression of MafA, Neurog3, Pdx1 and Pax4, master regulators of endocrine differentiation. More recently, human pancreatic organoids derived from ductal CD133^+^ cells could be differentiated into insulin^+^ cells (at a frequency of ∼4.6%) just by *in vitro* treatment with transcribed Neurog3 mRNA and differentiation media (Koblas et al., 2019). Characterization of liver and pancreas organoid-initiating cells in mice showed that these cells are phenotypically and functionally similar and express MIC1-1C3^+^/CD133^+^/CD26^-^ (Dorrell et al., 2014). Organoids derived from these ductal cells can differentiate into insulin expressing cells (at a frequency of ∼5–22%) following tricistronic adenoviral administration of MafA, Neurog3 and Pdx1. These insulin^+^ cells showed transcriptional identity partially overlapping with murine β cells, but with retained expression of many off-target non-β cell genes (Dorrell et al., 2014). In summary, pancreatic ductal-derived organoids have proven their potential for β cell replacement therapies although still the efficiency of organoids to differentiate into endocrine lineages is limited. A more refined reprogramming/differentiation methodology and a subpopulation of ductal cells that could produce an unlimited source of multipotent cells to generate endocrine cells is still needed (Figure 1).

To note, the media composition of the above studies comprises factors shown to be important in endocrine differentiation during development [e.g., FGFs (FGF10), EGF, RA] (Gittes, 2009; Santosa et al., 2016). These factors might also play a role in the differentiation of adult ductal organoids either potentiating or hindering the process, a field that deserves further investigation.

### Procr Positive Islet Derived Organoids

Finally, protein C receptor (Procr) is a surface protein that has been reported to mark stem cells in several adult tissues (Balazs et al., 2006; Iwasaki et al., 2010; Wang et al., 2015; Yu et al., 2016; Zhou et al., 2016; Fares et al., 2017). Interestingly, a recent comprehensive single cell RNA-seq analysis of murine islets identified a novel Procr^+^ population (Wang et al., 2020). Procr^+^ cells were characterized by a transcriptional signature indicative of epithelial-to-mesenchymal transition, lacked expression of terminally differentiated endocrine and exocrine markers, and were able to form islet-like organoid-like spheroids *in vitro*. Islet-like derived organoids could be long-expanded *in vitro* and upon co-culture with endothelial cells differentiated into endocrine cells with a remarkable high efficiency: after 1 month, up to 80% of cells expressed insulin. *In vivo*, long-term cultured organoids restored glucose homeostasis in streptozotocin-induced T1D mice (Wang et al., 2020). (Figure 1).

### Limitations and Future Perspectives

Several reasons could explain the limited endocrine differentiation capacity of organoids:

First, organoid maintenance media has been established based on the intestinal organoid media with minimal modifications (Fujii et al., 2019), therefore it is possible that a modified media that best mimics pancreatic progenitors’ requirements will influence positively their expansion, self-renewal and endocrine differentiation capacity. The stem cell/progenitor niche concept, initially proposed by Raymond Schofield in 1978, defines niches as compartments that are conductive for the maintenance of definitive stem cell properties (Schofield, 1978). The niche thus represents a defined anatomical compartment that provides signals to stem cells in the form of secreted and cell surface molecules to control the rate of stem cell proliferation, determine the fate of stem cell daughters and protect stem cells from exhaustion or death. Such a niche has not yet been identified in the adult pancreas. However, we know that through pancreas development the neuronal, mesenchymal and endothelial compartments are key for the proper maintenance and differentiation of embryonic progenitors into exocrine and endocrine lineages (Miralles et al., 1999; Lammert et al., 2001; Gittes, 2009; Borden et al., 2013). Potentially, complex organoid co-cultures with neuronal, mesenchymal and endothelial cells could shed light into the proper settings to mimic a progenitor niche and improve differentiation potential as it has been already proven in pluripotent stem cell cultures (Aghazadeh et al., 2021).

Second, the low regenerative capacity of the adult pancreatic tissue suggests that if a resident progenitor exists, it should be of really low abundance. Thus, not all ductal cells may bear progenitor capacity nor show similar organoid formation ability. Therefore, a better understanding of ductal heterogeneity could highlight ductal subpopulations to be studied in organoid culture and assess their endocrine differentiation capacity (Hendley et al., 2021).

Third, ductal-derived organoids could have a limited endocrine differentiation capacity due to epigenetic features. A better understanding of the differences between embryonic progenitors and organoids may identify an epigenetic brake to be modulated for proper endocrine differentiation.

Fourth, extracellular matrix (ECM) components might be crucial *in vitro,* as they are *in vivo*, for the proper morphogenesis and differentiation of the pancreatic lineages. Current organoid systems mostly rely on intrinsic or extrinsic biochemical signals (i.e., growth factors) and cell-cell interactions to control stem cell fate, but there are important elements to consider including the ECM and biophysical signals that are still unknown. This is because current *in vitro* models rely on animal-derived Matrigel which contains laminin, collagen IV, entactin, heparan sulfate, and growth factors (Rezakhani et al., 2021) in proportions that are difficult to manipulate. A controlled matrix rather than standard Matrigel could be key for organoid-to-β-cell differentiation.

Finally, the above published studies did not analyze the possible presence of endocrine cells in the primary ductal cultures. In this scenario, it cannot be discarded that some of the insulin producing cells observed at the end of the protocol were present in the cultures from the beginning. Further studies where the absence of insulin expressing cells in the original preparation is carefully ratified prior to organoid formation are still required to properly validate and estimate the efficiency of ductal to β cell transdifferentiation.

## Normal Tissue Derived Organoids for Cancer Research

### Ductal-Derived Organoids

**FIGURE 2:**
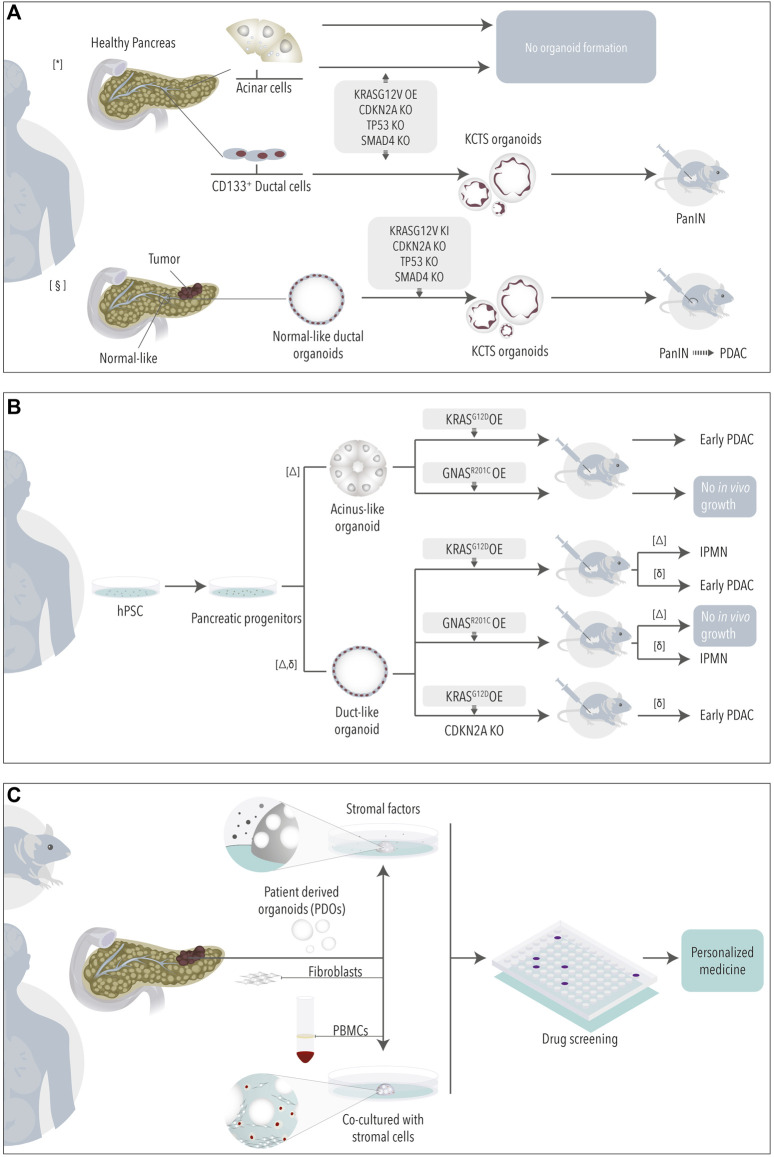
Organoids for cancer research. Non-tumoral organoids derived from healthy or normal-like tissue adjacent to tumor **(A)** or from pluripotent stem cells **(B)** have been engineered to study the role of PDAC driving mutations. **(C)** Tumoral organoids in mono- or co-cultures are used as model for drug testing. * (Lee et al., 2017) § (Seino et al., 2018) Δ (Huang et al., 2021), δ (Breunig et al., 2021).

Human healthy ductal derived organoids have also been used to model pancreatic cancer. Introduction of PDAC driver mutations in normal organoids *via* CRISPR-Cas9 or overexpression vectors has been used to study PDAC initiation and progression (Lee et al., 2017; Seino et al., 2018). Lee and colleagues (Lee et al., 2017) engineered organoids derived from healthy CD133^+^ ductal cells to express mutant *KRAS* ^G12V^ (K), and deleted *CDKN2A* (C), *TP53* (T), and *SMAD4* (S) (KCTS organoids). Following orthotopic implantation, the mutant organoids gave rise to early pancreatic lesions (PanINs), but these did not progress to PDAC. Differently, KCTS organoids developed by Seino *et al.* generated *in vivo* lesions comprising the full histological transformation from PanINs to invasive PDACs (Seino et al., 2018) (Figure 2A). Instead of healthy pancreata, Seino *et al.* used ductal cells isolated from “normal-like” regions adjacent to tumor tissue. Additionally, the two studies differed in the methodology to introduce *KRAS* ^G12V^ (overexpression *vs*. knock-in), the culture media used to establish the organoids, and the *in vivo* implantation model (orthotopic *vs*. subcutaneous). Whether some or all of these factors influenced the results deserves further investigation and encourages working on the development of standardized models. In the meantime, it is tempting to think that the origin of ductal cells (healthy *vs*. normal-like, CD133^+^ *vs*. bulk) may influence organoids’ nature and that microenvironmental cues (culture media, site of implantation) will need to be adjusted in search of more representative models.

### Acinar-Derived Cultures

The above studies employed organoids derived from ductal cells to study PDAC formation. However, the cell of origin of PDAC and its implication on the progression and biology of tumors are still a matter of debate. Several studies have shown that both acinar and ductal cells can give rise to tumors in genetic mouse models (Guerra et al., 2007; Von Figura et al., 2014; Bailey et al., 2016; Ferreira et al., 2017; Lee et al., 2019; Flowers et al., 2021). Some of these studies have recently highlighted that mouse PDAC tumors arising from either acinar or ductal cells show differences at the transcriptomic and phenotypic levels (Ferreira et al., 2017; Lee et al., 2019; Flowers et al., 2021). The existence of different cells of origin shall, in part, explain the great heterogeneity observed in the PDAC human scenario (Raphael et al., 2017; Collisson et al., 2019; Backx et al., 2021a; Espinet et al., 2021). Thus, models where the impact of mutations can be studied in both acinar and ductal human cells may help us to better understand patient tumor complexity. Unfortunately, acinar cells fail to grow organoids (Seino et al., 2018) even when engineered to express the main PDAC mutations (Lee et al., 2017). In general, acinar cells are refractory to *in vitro* culture, even if successfully isolated, as they rapidly undergo transformation into ductal-like cells (acinar-to-ductal metaplasia) (Houbracken et al., 2011; Wollny et al., 2016).

### iPSC-Derived Organoids

An approach to overcome the above limitation consists in inducing the differentiation of human PSCs (hPSCs) into acinus- and ductal-like exocrine organoids (Huang et al., 2015; Hohwieler et al., 2017). To note, hPSCs have been widely studied also as a source to obtain endocrine cells *via* differentiation, a topic that will not be discussed here as it has been extensively reviewed elsewhere (Melton, 2021). hPSCs exocrine differentiation requires a sequential protocol in which ESCs are first differentiated into pancreatic progenitor-like cells and then into acinus or ductal-like cells (Huang et al., 2015; Hohwieler et al., 2017). Using this approach, Huang and colleagues recently showed that PDAC driver mutations lead indeed to cell-lineage-specific phenotypes (Huang et al., 2021). While *KRAS* ^G12D^ in acinus-like organoids gave rise mainly to early PDAC lesions upon orthotopic transplantation, *KRAS* ^G12D^ duct-like organoids were less efficiently engrafting and mainly developed IPMN-like lesions (Figure 2B). Differently, if presenting with a *GNAS* ^R201C^ mutation neither acinus- nor ductal-like organoids engrafted *in vivo* (Huang et al., 2021). Another study, by Breunig and colleagues, in which ESCs were differentiated into pancreatic ductal-like organoids (PDLOs) confirmed the tropism of these different mutations, although with different results (Breunig et al., 2021). In this study, *KRAS* ^G12D^ in PDLOs resulted in early PDAC lesions upon orthotopic engraftment while *GNAS* ^R201C^ PDLOs developed IPMNs-like structures (Figure 2B).

### Limitations and Future Prospective

Differentiated hPSCs are a valuable model to study early mutagenic events in human cells. However, it is important to investigate the reasons behind divergent results obtained in, otherwise, similar studies. If possible, unifying protocols and definitions of hPSC-derived exocrine organoids may help conciliate the appearance of contrasting results towards a better understanding of early mutagenesis in human PDAC. Additionally, the existence of acinar and ductal heterogeneity, recently revealed by single cell sequencing technologies (Arda et al., 2016; Muraro et al., 2016; Enge et al., 2017; Martens et al., 2021; Tosti et al., 2021), will need to be considered in future studies. In promising latter single cell analyses of hPSC derived ductal-like organoids, Wiedenmann, Breunig and colleagues have shown the appearance of different types of ductal-like cells that share characteristics with ductal subsets identified in normal pancreas (Huang et al., 2021; Wiedenmann et al., 2021). This suggests that certain relevant ductal heterogeneity can be recapitulated in the hPSC model. Although scRNA-sequencing of hPSC-derived acinus-like organoids also depicted certain heterogeneity (Huang et al., 2021), overall, acinus-like organoids resembled fetal rather than mature pancreatic acini. Thus, the relevance of this observed “acinar heterogeneity” in the adult pancreas context might be questionable. Using adult mouse pancreas studies showed that only a small subset of acinar cells have long term proliferative capacity *in vivo* (Wollny et al., (2016) *.* While these results suggest exciting functional differences in the adult acinar compartment, the study of the role of these and other acinar cells in the human and cancer scenario is still hindered by the lack of stable acinar *in vitro* models. Adaptation of the current culture conditions used to grow acinus-like organoids derived from hPSCs or from adult acini directly might result in improved acinar organoid formation. The big challenge is the fact that acinar cells rapidly lose their acinar identity upon injury or stress to move towards a ductal-like phenotype. The simple isolation of acinar cells from adult tissue might thus induce dedifferentiation of acinar cells. New studies analyzing these early events *in vitro* might help us to find the proper conditions required by acinar cells to retain their identity during longer experimental times (Backx et al., 2021b).

## Pancreatic Cancer Derived Organoids

Following the work in other tumor types, in 2015 Clevers and Tuveson’s groups described the first protocol to establish organoids from mouse and human PDAC tissue (Boj et al., 2015). Since then, many groups have used or adapted the protocol to generate small biobanks of patient derived organoids (PDOs) with the aim to use them as avatar models to define more personalized therapies. When compared to patient derived xenografts models (PDXs), PDOs are generated faster and allow drug testing on a larger scale with lower cost (Tuveson and Clevers, 2019). Additionally, PDOs can be generated from little material obtained from fine needle biopsies, allowing the generation of models from non-resectable patients and from metastases. Thus, PDOs not only represent a model to study the biology of tumor cells, but also hold the promise of being a platform to screen more effective drugs in a personalized and relevant-time manner. A recent comparison of the treatment of PDOs *versus* PDXs showed similar responses in the two models supporting the use of *in vitro* organoids as surrogate model (Frappart et al., 2020). Recent comparisons of the response of PDOs *versus* the actual patient response further encourage the study of organoids in the clinical setting (Frappart et al., 2020; Grossman et al., 2021).

### Pancreatic Cancer Organoids for Precision Oncology: Promises and Considerations

Two major transcriptomic epithelial subtypes have been described in PDAC after a collection of studies analyzing primary tumor samples: the classical and the basal-like subtypes (Martens et al., 2019). Tumors with a classical epithelial subtype show expression of genes characteristic of pancreatic progenitor cells and tend to be differentiated with better outcome. The basal-like epithelial subtype associates to more dedifferentiated and advanced tumors and correlates with worse outcome and lesser response to treatment (Aung et al., 2018). The emergence of PDAC PDOs offers a practical platform to study the biology of these two epithelial subtypes and to investigate subtype specific vulnerabilities. Additionally, efforts are done to generate transcriptomic signatures according to the response of different PDOs *in vitro*, that can be used to predict patient responses (Tiriac et al., 2018; Nicolle et al., 2020; Nicolle et al., 2021). The power of these signatures, some of which are currently being tested in clinical trials, will need to be carefully evaluated to find the best predictive markers.

Some studies have adapted the original PDAC-organoid media composition (Boj et al., 2015) to generate PDOs (Huang et al., 2015; Seino et al., 2018; Driehuis et al., 2019; Krieger et al., 2021). Although finding a consensus media to unify and standardize PDO growth across laboratories seems desirable, defining a unique media might be, in this case, delicate. Different tumor organoid subtypes present distinct dependency towards external soluble factors (Seino et al., 2018) likely reflecting existing differences of their tumor microenvironments (Nicolle et al., 2017; Maurer et al., 2019; Grünwald et al., 2021). Additionally, *in vivo* treatment with cytokines or implantation of organoids in different *in vivo* niches has shown to change the transcriptome of PDAC cells highlighting the plasticity of these cells under the influence of external factors (Miyabayashi et al., 2020; Tu et al., 2021). In line, the recent study by Raghavan and colleagues described that PDOs acquire culture-specific transcriptomic programs that are not present when the cells are in the tumor (Raghavan et al., 2021). More importantly, different media conditions (rather than 3D *versus* 2D structural changes) resulted in transcriptomic changes and influenced drug response of tumor cells (Raghavan et al., 2021). While we desire to move towards a standardized model as a tool for personalized medicine, these new observations question if we rather need to move towards fully personalized models first.

To add to this complex scenario, chemotherapeutic treatment has also been described to induce plasticity of PDAC tumor cells with transition from basal to classical subtype or *vice versa* having been observed (Chan-Seng-Yue et al., 2020; Hwang et al., 2020). This suggests that cancer cells undergo transcriptional subtype switching to adapt to the administered drug. In addition, Peschke, Jakubowsky and colleagues have recently reported a classical to classical case after FOLFIRINOX treatment (Peschke et al., 2022) where PDOs derived from the pre- and the post-treated tumors showed remarkable differences to drug responses *in vitro* despite sharing genetic drivers and being both classified as classical (Peschke et al., 2022). These results indicate that a transcriptional subtype switch does not occur always, and that drug adaptation can be achieved by other, unknown, mechanisms. Together, the use of current transcriptomic classifications to predict treatment response might need to be revised in the post-treatment scenario. Further knowledge needs to be acquired to understand how the plasticity-emerged subtypes compare to the original subtypes defined in the naïve treatment scenario and how this impacts prediction of treatment response. Whenever possible, performing longitudinal functional assays with PDOs obtained pre- and post-treatment will be a valuable tool for a precision oncology approach.

## Final Notes

Organoids are a very powerful tool to understand pancreatic biology and to help us to develop valuable therapies such as tissue transplantation and personalized cancer treatment. As for Spiderman, for organoids “*with great potential comes great prospects*”. Notwithstanding the above message, organoids are heterogeneous in shape and size; moreover, the absence of blood supply and interactions with other pancreatic cell types and non-pancreatic tissues limits their potential to date. For this, we need to standardize organoid cultures, including co-cultures with other cells (mesenchymal, endothelial, neuronal and immune cells), and to improve cell maturation to more faithfully model *ex vivo* the actual *in vivo* gland. In the meantime, while we continuously move towards better models, we should still acknowledge the value of standard models. Likely, the combination of models rather than a unique one will help us answer our questions.

**BOX 1 | Pancreatic 3D Cultures**

**Aggregates:** Cell aggregates are formed by artificially forcing cells to grow together in culture. Most common methods include use of low attachment culture plates, agarose micro-wells or hanging-drop cultures that pool cells together (Cavallari et al., 2007; Hilderink et al., 2015; Ware et al., 2016; Zuellig et al., 2017; Wassmer et al., 2020).

**Spheroids:** Spheroids are spherical cellular units that are generally cultured as free-floating aggregates with no matrix component and are of low complexity. Spheroids can be generated from immortalized cell lines, primary cells or fragments of tissue and, as such, their viability is limited, as they do not contain a progenitor phenotype. Spheroids develop a necrotic core as they grow in size and possess no or limited tissue structure and a less representative tissue architecture. Sphere-forming assays have been widely used in 1) stem cell biology to assess the self-renewal and differentiation potential of a particular cell type 2) *in vitro* models to investigate solid tumors.

The first spheroids used to investigate stem cells were developed to investigate the presence of adult neuronal stem cells (neurospheres) (Reynolds and Weiss, 1992). These assays have been adopted to investigate stem cells and progenitors in a variety of tissues. A suffix is usually added to identify the tissue of origin of the spheroids, such as, mammary gland-derived mammospheres (Dontu et al., 2003), pancreas-derived pancreatospheres (Kerr-Conte et al., 1996; Seaberg et al., 2004; Smukler et al., 2011) and prostate-derived prostatospheroids (Rajasekhar et al., 2011).

**Organoids:** Organoids have been used as 3D cell culture models to study adult progenitors and tumor biology since 2009 when they were first described by Clevers’ laboratory (Sato et al., 2009). Organoids are more complex than spheroids and are three-dimensional cell cultures that incorporate some of the key features of the organ of origin. These *in vitro* culture systems contain a self-renewing stem cell population that differentiates into multiple, organ-specific cell types that exhibit spatial organization like the corresponding organ and can recapitulate some functions of the organ providing a highly physiologically relevant system.

Organoids are derived from one or a few adult stem cells of a tissue, from embryonic stem cells or from induced pluripotent stem cells. Typically, they require a scaffold to grow, such as Basement Membrane Extract (BME), Matrigel or extracellular matrix components.

Tumor-derived organoids from patients have opened a window of opportunity to investigate more complex tumor microenvironment, disease modelling and personalize medicine therapies.

## Glossary

2D: 2-dimensional
3D: 3-dimensional
ALDH: Aldehyde Dehydrogenase
BME: Basal membrane extract
BMP: Bone morphogenetic protein
CD26: Cluster of differentiation 26, Dipeptidyl peptidase-4
CD133: Cluster of differentiation 133, Prominin-1
CDKN2A: Cyclin dependent kinase inhibitor 2A
ECM: Extracellular Matrix
EGF: Epidermal growth factor
EpCAM: Epithelial Cell Adhesion Molecule
ESC: Embryonic Stem Cell
FGF: Fibroblast growth factor
GNAS: GNAS complex locus
IPMN: Intraductal papillary mucinous neoplasm
iPSC: Induced Pluripotent Stem Cell
KRAS: KRAS proto-oncogene, GTPase
Krt19: Cytokeratin-19
Lgr5: Leucine-rich repeat-containing G-protein coupled receptor 5
MafA: MAF bZIP transcription factor A
MIC1-1C3: Oval Cell Marker MIC1
MUC1: Mucin 1, CD227
Neurog3: Neurogenin 3
PanIN: Pancreatic intraepithelial neoplasia
Pax4: Paired box 4
PDAC: Pancreatic Ductal Adenocarcinoma
PDLO: Pancreatic ductal-like organoids
PDO: Patient derived organoids
PDX: Patient derived xenografts
Pdx1: Pancreatic and duodenal homeobox 1
PP: Pancreatic Polypeptide
Procr: Protein C receptor
RA: Retinoic Acid
RSPO-1: R-spondin1
SMAD4: SMAD family member 4
Sox9: SRY (Sex determining region Y)-box9
T1D: Type 1 Diabetes
TGFβ: Transforming growth factor beta 1
TP53: Tumor protein p53
TSQ: Methoxy-8-p-toluenesulfonamido-quilone
